# MAKA-Map: Real-Valued Distance Prediction for Protein Folding Mechanisms via a Hybrid Neural Framework Integrating the Mamba and Kolmogorov–Arnold Networks

**DOI:** 10.3390/biom16020194

**Published:** 2026-01-27

**Authors:** Benzhi Dong, Yumeng Hua, Chang Hou, Dali Xu, Guohua Wang

**Affiliations:** 1 College of Computer and Control Engineering, Northeast Forestry University, Harbin 150040, China; nefudbz@nefu.edu.cn (B.D.); hym@nefu.edu.cn (Y.H.); houchang@nefu.edu.cn (C.H.); 2 Faculty of Computing, Harbin Institute of Technology, Harbin 150001, China

**Keywords:** protein, real-valued distance prediction, Mamba, KAN

## Abstract

Real-valued inter-residue distance maps provide essential spatial information for understanding protein folding mechanisms and guiding downstream applications such as function annotation, drug discovery, and structural modeling. However, existing prediction methods often struggle to capture long-range dependencies and to maintain topological consistency across different structural scales. To address these challenges, we propose a novel prediction framework that integrates a Mamba architecture, based on a selective state space model, to effectively model global interactions, and incorporates the Kolmogorov–Arnold Network (KAN) to enhance nonlinear structural representation. Extensive experiments on standard benchmark datasets, including CASP13, CASP14, and CASP15, demonstrate prediction accuracies of 86.53%, 85.44%, and 82.77%, respectively, outperforming state-of-the-art approaches. These results indicate that the proposed framework substantially improves the fidelity of real-valued distance prediction and offers a promising tool for downstream structural and functional studies.

## 1. Introduction

In recent years, significant advancements have been made in drug–target affinity (DTA) prediction research [1,2], driven largely by the integration of protein structural information [3]. Numerous studies have demonstrated that incorporating protein structural data effectively enhances the accuracy of DTA predictions compared with traditional sequence-based methods [4,5]. In particular, the use of real-valued distance maps between protein residues enables a detailed characterization of the target protein’s spatial conformation, facilitating more accurate predictions of binding affinities for small-molecule ligands.

Models such as CASTER-DTA exemplify the advantages of integrating real-valued residue distance information into map construction and feature encoding [6]. Experimental results across several benchmark datasets consistently indicate that graph neural networks leveraging this structural information significantly outperform those relying solely on sequence-based data. Furthermore, removal of structural features based on real-valued distance maps leads to a substantial decline in model performance, underscoring their critical role in structure-oriented tasks, such as DTA prediction.

The contribution of real-valued distance maps to DTA prediction is primarily manifested in two aspects. First, they precisely capture the geometric relationships among protein residues, enabling models to better reflect physically plausible interaction patterns. Second, this structural representation provides essential support for developing geometrically equivariant models, significantly enhancing adaptability and generalizability across diverse protein conformations. Consequently, constructing high-quality real-valued distance maps has emerged as a vital strategy for improving model accuracy in drug–target affinity prediction, thereby offering more precise and reliable computational tools for drug discovery and development.

With the rapid growth of protein sequence databases [7,8] and the advancement of deep learning technologies, neural networks [9,10,11] have become predominant in the prediction of real-valued distance maps for proteins. Representative methods include PDNET [12], which integrates deep residual networks for distance prediction and enables improved 3D protein structure construction. Additionally, trRosetta [13] expands the multi-task paradigm by concurrently predicting inter-residue distances and orientation angles. Meanwhile, SDP [14] enhances prediction accuracy for longer distances and extended sequence intervals by simplifying the feature space and refining the network architectures. More recently, MF-ProtDisMap [15] integrates MSA-derived coevolutionary features with protein language model representations, and further enhances real-valued distance prediction through multi-feature fusion and diffusion-based representation learning.

Despite these advances, existing methods still struggle to capture medium- and long-range dependencies adequately and to maintain global topological consistency, leaving substantial room for improvement in both the overall correlation between predicted and actual real-valued distance maps and local accuracy. To address these challenges, we propose a novel deep learning framework, MAKA-Map, which is built upon a residual encoder-decoder architecture. Our approach innovatively integrates the Mamba architecture [16], based on state space modeling, with the Kolmogorov–Arnold Network (KAN) [17]. Specifically, the Mamba module effectively captures medium- to long-range sequence information, while the KAN enables the modeling of complex nonlinear structural relationships via learnable nonlinear activation functions. This synergy facilitates multi-scale interaction representation and preserves topological coherence, thereby enhancing consistency between local features and global structural information, while significantly improving the model’s capacity to represent higher-order protein structural features. Evaluations on benchmark datasets (CASP13, CASP14, and CASP15) demonstrate the superior predictive performance of our method, underscoring its strong potential and broad applicability in protein structure modeling.

## 2. Materials and Methods

### 2.1. Datasets

The training data used in this study originate from two publicly curated, non-redundant, and openly accessible benchmark datasets that provide protein structures together with precomputed multiple sequence alignments (MSAs). The first source is the DeepCov dataset [18], from which all 3456 preprocessed protein chains were included. The second source is the trRosetta training dataset [13], which contains a large collection of protein structures with accompanying MSAs; from this dataset, 5802 protein samples were retained. Protein entries were selected using simple yet task-oriented criteria. Specifically, we preserved samples with available precomputed MSAs that were compatible with our feature-generation pipeline and whose sequence lengths fell within the range of approximately 50–500 amino acids to satisfy the input size constraints of our model architecture. In total, 9258 protein samples were assembled to construct the training dataset.

This dataset excludes sequences with detectable homology to test sets, thereby preventing overlap between our training data and test sets. In all cases, we used the MSAs provided by the original datasets and extracted derived features such as position-specific scoring matrices (PSSMs), coevolutionary coupling scores, and sequence conservation measures. All data were standardized and reformatted to ensure consistency and comparability of input features across the two sources.

For model evaluation, three widely recognized benchmark datasets in the protein structure prediction community, namely CASP13 [19], CASP14 [20], and CASP15 [21], were employed as held-out test sets. Each CASP edition contains targets with distinct levels of difficulty and structural novelty, ranging from proteins with well-characterized traditional folds (CASP13) to more diverse and challenging targets with higher sequence variability and lower homology support (CASP14 and CASP15). All CASP targets were processed using the same feature-generation pipeline as the training data to ensure fair and consistent evaluation.

### 2.2. Feature Selection

#### 2.2.1. MSA-Derived Feature Representation

In this study, a variety of features were used as key inputs to the model, enabling the capture of evolutionary relationships and potential structural information from protein sequences. These features exhibit strong correlations with protein structure and have been widely adopted by related studies, as summarized below:

- Coevolutionary signals: Predicted using CCMpred [22] to capture the co-evolutionary strength between all residue pairs in a multiple sequence alignment (MSA), reflecting their potential synergistic mutations over evolutionary time;
- FreeContact feature: A contact prediction tool based on co-evolutionary information that provides estimates of residue-residue interaction strengths to aid in modeling possible physical contacts [23];
- Position-specific scoring matrix (PSSM): Derived from MSA calculations, it describes the probability distribution of amino acid occurrences at each position, capturing evolutionary conservation;
- Contact potentials: Covariant frequency information inferred from MSA, combined with sequence weighting, used to reveal potential structural energy constraints between residue pairs [24,25];
- Shannon entropy of the alignment column: A metric quantifying sequence variability across alignment positions, used to assess sequence diversity through information entropy [24,25].

The integration of these features comprehensively captures the structural patterns and evolutionary properties of protein sequences at multiple levels, providing richs, high-quality input for the deep learning model and thus effectively enhancing its performance in predicting real-valued inter-residue distance maps.

#### 2.2.2. Feature-Space Independence Analysis

To evaluate the distributional differences between the training and test sets within the MSA-derived feature space, we conducted a quantitative similarity analysis based on protein feature representations. For each protein sample, we integrated multiple MSA-based features, including the position-specific scoring matrix (PSSM), sequence entropy, and coevolutionary coupling information obtained from CCMpred and FreeContact. To enable comparison across proteins of varying sequence lengths, summary statistics—including the mean, standard deviation, and selected quantiles—were computed for each feature, yielding fixed-length embedding vectors that map variable-length MSA information into a unified feature space.

Within this feature space, cosine distance was employed to quantify dissimilarity between protein samples, where smaller values indicate higher feature similarity and larger values reflect more pronounced distributional differences. To prevent information leakage from the test set during normalization, all feature vectors were standardized using Z-score normalization based exclusively on statistics derived from the training set.

We first computed the nearest-neighbor distances among training samples, denoted as $D_{\mathrm{train}\to \mathrm{train}}$, which serve as a baseline distribution characterizing intra-training-set feature similarity. In parallel, we calculated the distance from each test sample to its nearest neighbor in the training set, denoted as $D_{\mathrm{test}\to \mathrm{train}}$, to assess the relative positioning of test samples within the same feature space.

**Figure 1:**
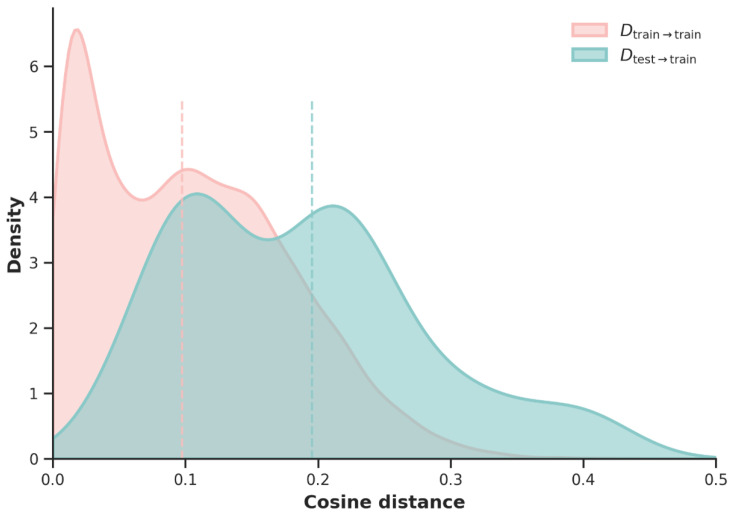
Distribution of nearest-neighbor cosine distances in the MSA-derived feature space.

As illustrated in Figure 1, the probability density distributions of $D_{\mathrm{train}\to \mathrm{train}}$ and $D_{\mathrm{test}\to \mathrm{train}}$ are shown by the pink and cyan curves, respectively. The pink curve represents the distribution of $D_{\mathrm{train}\to \mathrm{train}}$, while the cyan curve corresponds to $D_{\mathrm{test}\to \mathrm{train}}$. All density curves are normalized to facilitate comparison of distributional shapes. All density curves are normalized; therefore, the area under each curve does not represent sample size but is used solely for comparing distributional shapes. The distribution of $D_{\mathrm{test}\to \mathrm{train}}$ exhibits an overall positive shift relative to the training baseline, with a median value of approximately 0.196, compared to a median of approximately 0.098 for $D_{\mathrm{train}\to \mathrm{train}}$. This indicates that test samples generally reside at larger feature distances from training samples in the MSA-derived feature space. Furthermore, while $D_{\mathrm{train}\to \mathrm{train}}$ shows a degree of concentration near zero, the minimum value of $D_{\mathrm{test}\to \mathrm{train}}$ exceeds 0.05, suggesting the absence of test samples that are highly similar to the training data.

Overall, the test set is distributed beyond the baseline feature distribution of the training set, demonstrating a clear degree of distributional distinctiveness in the MSA-derived feature space. This analysis supports the validity of the training–test partition at the feature level and provides a reliable foundation for subsequent evaluation of the model’s generalization performance.

### 2.3. Architecture of MAKA-Map

To effectively process multidimensional protein features and improve the accuracy of real-valued distance prediction, we propose a novel hybrid deep neural network comprising three core modules: a Perceptive Encoder, a Structural Refiner, and a Prediction Decoder. Our architecture addresses key challenges inherent in residue-pair matrix representations, including modeling long-range dependencies, capturing complex inter-channel relationships, and enhancing higher-order structural representations. Specifically, the model integrates the state space-based Mamba architecture and the interpretable Kolmogorov–Arnold Network (KAN), enabling comprehensive improvements at the levels of residue-pair interaction, channel-wise semantic representation, and predictive mapping. Throughout this section, L denotes the length of the input protein sequence, and all residue-pair representations are formulated as L × L matrices.

First, the Perceptive Encoder efficiently models long-range dependencies in residue-pair matrices by incorporating the Mamba module. This module addresses the limitation of traditional convolutional receptive fields and enhances the model’s ability to capture the global relationship between residue pairs. Second, the Structural Refiner, positioned at the bottleneck of the backbone, incorporates the Kolmogorov–Arnold Network (KAN), which reconstructs complex structural relationships between residue-pair channels through explicit nonlinear mapping. This integration significantly enhances channel-level expressiveness and improves the consistency and interpretability of residue-pair prediction maps. Finally, the Prediction Decoder progressively integrates multi-scale encoded features using transposed and depthwise separable convolutions. This design enhances the modeling of local structural relationships while preserving the resolution of the residue-pair matrix. The decoder further applies a Softplus activation function to constrain the output to non-negative real-valued distances, aligning with the requirements of continuous regression tasks for protein residue-pair prediction.

**Figure 2:**
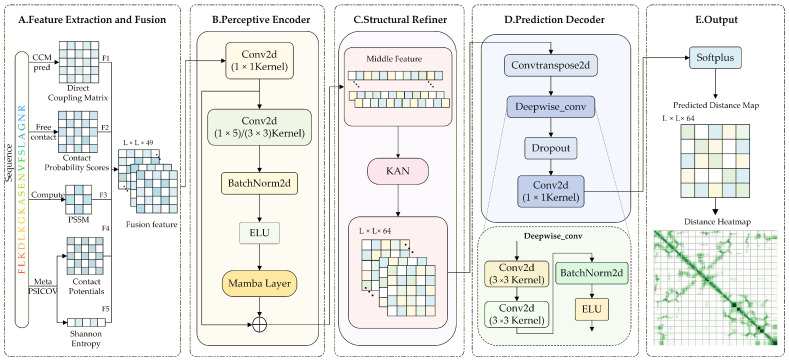
Overall architecture of the proposed MAKA-Map framework.

The model comprises three main sub-modules, as illustrated in Figure 2.

(1) Mamba-based Perceptive Encoder Module

**Figure 3:**
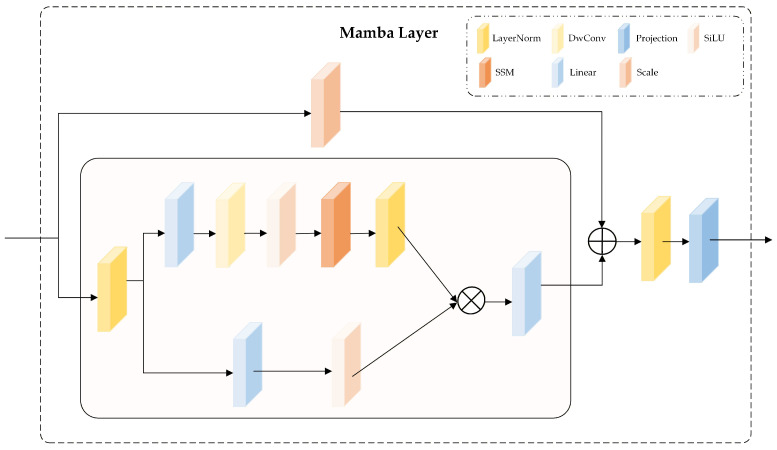
Structure of the Mamba module.

As illustrated in Figure 2B, the Perceptive Encoder Module utilizes a multi-scale residual encoder, designed to maintain a constant spatial resolution, thereby effectively capturing both local features and medium- and long-range dependencies. This encoder comprises 64 progressive residual stages, each incorporating alternating convolutional kernels (1×5 and 3×3), thereby expanding the receptive fields and increasing feature diversity. Critically, each stage integrates the Mamba architecture (Figure 3), substantially enhancing the network’s capacity to model medium- and long-range residue-pair interactions.

To facilitate efficient feature propagation and fusion across stages, the encoder employs skip connections. Specifically, at each stage, input features undergo convolutional transformations, nonlinear activations, and processing through the Mamba module before being combined with the original inputs via residual connections. This design promotes robust multi-scale feature integration, thereby stabilizing training and improving prediction accuracy. This mechanism facilitates efficient flow and fusion of feature information across scales, contributing to improved stability and accuracy in downstream predictions. The detailed architecture of the Mamba module is illustrated in Figure 3.

The input tensor has the shape (B,C,H,W), where *B* denotes the batch size, *C* the number of channels, *H* the height, and *W* the width. As illustrated in Figure 3, the inputs are first processed using LayerNorm, which standardizes features at each sequence position, thereby enhancing the stability of subsequent modeling.

The normalized inputs are then fed into a network module that incorporates a parallel branching structure to enhance modeling diversity while preserving state space modeling capabilities. As shown in Figure 3, the first branch captures nonlinear responses solely through a linear transformation followed by the SiLU activation. In the second branch, the input features are first expanded along the channel dimension via a linear transformation. They are then sequentially processed by depthwise convolution (DWConv), the SiLU activation function, and a state space model (SSM), and finally normalized using LayerNorm. The outputs of the two branches are fused via a Hadamard product and subsequently projected back to the original channel dimension through a linear mapping to produce the final output representation. The mathematical formulation is given in Equations (1)–(3):

(1) $$W_{1}=\mathrm{LayerNorm}\left(\mathrm{SSM}\left(\mathrm{SiLU}\left(\mathrm{DWConv}\left(\mathrm{Linear}(W_{\mathrm{in}})\right)\right)\right)\right),$$

(2) $$W_{2}=\mathrm{SiLU}\;\left(\mathrm{Linear}\;\left(W_{\mathrm{in}}\right)\right),$$

(3) $$W_{\mathrm{out}}=\mathrm{Linear}\;\left(W_{1}⊙W_{2}\right),$$

where ⊙ denotes the Hadamard (element-wise) product.

Following the above computation, the output is fused with the input feature map *x* via a residual connection with a learnable scaling factor *s*, thereby preserving the original features and enhancing deep representations. This fusion process is defined in Equation (4):

(4) $${\tilde{M}}^{\;l}=W_{\mathrm{out}}+s\cdot x,$$

where ${\tilde{M}}^{\;l}$ denotes the intermediate output at layer *l*.

Subsequently, the output tensor undergoes another LayerNorm operation, followed by a linear projection to the target output dimension. This process is formally defined in Equation (5):

(5) $$M_{\mathrm{out}}^{\;l}=\mathrm{Projection}\;\left(\mathrm{LayerNorm}\;\left({\tilde{M}}^{\;l}\right)\right).$$

Finally, the output tensor is transposed and reshaped into a distance map with the structure $\left(B,C_{\mathrm{out}},H,W\right)$.

This process enables the model to more accurately capture protein residue interactions, thereby effectively modeling long-range spatial dependencies. As a result, it improves the accuracy of distance map prediction while reducing both the number of parameters and the overall computational complexity.

(2) Structural Refiner Based on Kolmogorov–Arnold Networks

To further enhance higher-order feature modeling, a KAN-based structural inference mechanism is incorporated into the Structural Refiner, with the detailed architecture illustrated in Figure 2C. The module first reshapes and rearranges the spatial feature-map channels into one-dimensional sequences. Subsequently, higher-order nonlinear transformations are performed using a dedicated KAN Linear layer, which leverages the Kolmogorov–Arnold Network (KAN) for feature interpolation and adaptive function fitting. A dynamic mesh adjustment mechanism further refines this adaptive fitting, significantly improving the model’s sensitivity to local-global structural consistency and thereby enhancing the topological accuracy of the predicted distance maps.

**Figure 4:**
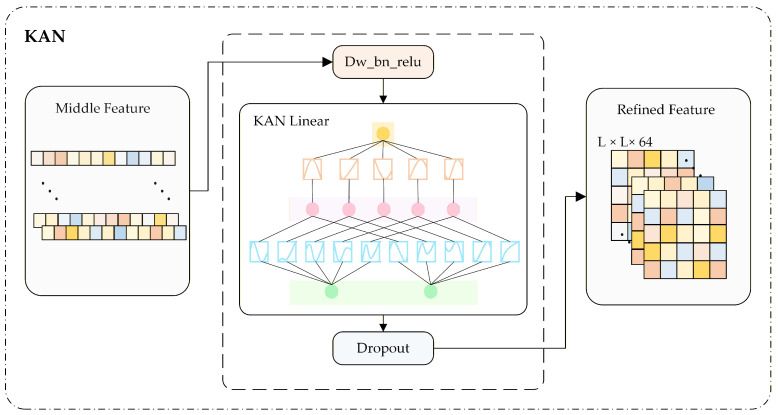
Architecture of the Kolmogorov–Arnold Network (KAN).

The Kolmogorov–Arnold Network (KAN) architecture, inspired by the Kolmogorov–Arnold representation theorem, replaces conventional linear mappings with adaptive nonlinear transformations, achieving both greater efficiency and enhanced interpretability. A detailed illustration of the KAN architecture is shown in Figure 4.

First, the input features $X\in R^{B\times C\times H\times W}$ are reshaped into a sequence of shape (B,H×W,C). Local information is extracted using a lightweight convolutional block comprising a depthwise separable convolution (DWConv), followed by batch normalization (BatchNorm) and ReLU activation. The features are then nonlinearly mapped along the channel dimension using KAN, which does not rely on fixed activation functions. Its structure is illustrated in Figure 4, which depicts the nonlinear mapping of inputs via a matrix of learnable activation functions constructed from B-spline basis functions. The computational procedure is formally defined in Equation (6):

(6) $$\mathrm{KAN}\left(Z\right)=\phi_{0}Z,$$

where *Z* denotes the input feature and $\phi_{0}$ represents a matrix of learnable activation functions. Its explicit formulation is provided in Equation (7):

(7) $$\phi_{0}=\left(\begin{array}{cccc} \phi_{1,1} & \phi_{1,2} & \cdots & \phi_{1,n_{\mathrm{out}}}\\ \phi_{2,1} & \phi_{2,2} & \cdots & \phi_{2,n_{\mathrm{out}}}\\ \vdots & \vdots & \ddots & \vdots \\ \phi_{n_{\mathrm{in}},1} & \phi_{n_{\mathrm{in}},2} & \cdots & \phi_{n_{\mathrm{in}},n_{\mathrm{out}}} \end{array}\right).$$

Each $\phi_{q,p}$ denotes a learnable nonlinear activation function. The Kolmogorov–Arnold Network models complex nonlinear relationships by mapping input features through this nonlinear structure, thereby eliminating the need for a conventional linear weight matrix. Additionally, KAN effectively captures complex, nonlinear contact patterns in proteins. It offers greater expressive power and interpretability than traditional linear mappings, thus compensating for the limitations of conventional neural networks in structural modeling. By integrating stability and interpretability with expressive capacity, KAN enables more physically and biologically meaningful feature reconstruction, thereby improving the model’s ability to capture complex residue–residue interactions.

(3) Real-Valued Prediction Decoder

As illustrated in Figure 2D, the Real-Valued Prediction Decoder employs an asymmetric N−1-layer design, where *N* represents the number of encoder stages. This decoder performs progressive upsampling using transposed convolutions and integrates multi-stage depthwise separable convolutions alongside cross-scale skip connections to incrementally reconstruct structural details. Finally, a 1×1 convolution followed by a Softplus activation function outputs the real-valued inter-residue distances, thereby completing the prediction process. The final output format is illustrated in Figure 2E.

### 2.4. Model Training and Prediction Process

To enable accurate real-valued distance prediction, the model is designed to maintain a consistent spatial resolution. Unlike traditional binary classification frameworks, the prediction stage does not involve complex categorization. Instead, continuous distance values are retained, enhancing the model’s representational capacity and downstream flexibility.

Feature extraction plays a critical role in the proposed model. To effectively capture sequence-structure coupling information, several well-established tools are employed for feature computation, as illustrated in Figure 2A. Co-evolutionary features are extracted using CCMpred and FreeContact, which respectively estimate the strength of synergistic mutation between residue pairs and their physical contact potentials. PSSM features are derived from one-hot encoded multiple sequence alignment (MSA) data and enhanced via a sample reweighting strategy to improve the statistical reliability of the resulting frequency matrix. Additionally, MetaPSICOV is used to compute evolutionary conservation and sequence statistics, from which higher-order features such as contact potentials and Shannon entropy across alignment columns are derived. All features are derived from MSA results and uniformly formatted as two-dimensional residue-pair feature matrices, serving as inputs for subsequent structure prediction tasks.

To ensure an architecture-level fair comparison, all baseline models are re-implemented under the same unified feature set as MAKA-Map. We acknowledge that trRosetta and MF-ProtDisMap rely on feature pipelines—such as orientation distributions in trRosetta, as well as MSA-based coevolutionary features and protein language model representations in MF-ProtDisMap—that are not present in our unified setting. Therefore, their performance under our unified features does not replicate the magnitudes reported in their original publications. This deviation reflects their sensitivity to input feature dependencies rather than degradation caused by our re-implementation. PDNet and SDP, in contrast, show stable performance comparable to their originally published results under the same unified feature set. The unified-feature protocol is intended to isolate architectural differences under strictly identical input information. All models are trained and evaluated using identical data splits, optimization strategies, and evaluation metrics, ensuring consistent and reproducible comparisons.

In this module, bottom-up features are progressively fused by the decoder while preserving spatial dimensions across all layers. The final layer applies a 1×1 convolution for channel compression, resulting in a single-channel, real-valued distance map.

To quantify the discrepancy between predicted values and ground-truth labels, a customized loss function based on the inverse hyperbolic cosine function is employed. This loss penalizes inverse residual errors with a smoothing effect that varies with distance, as formally defined in Equation (8).

(8) $$L_{\mathrm{inv-log-cos}h}=\frac{1}{N}\sum_{i,j}\log\cosh\left(\frac{100}{{\hat{D}}_{i,j}+\varepsilon }-\frac{100}{D_{i,j}+\varepsilon }\right),$$

where $D_{i,j}$ denotes the ground-truth distance, ${\hat{D}}_{i,j}$ is the model prediction, and ε is a small constant added to avoid division by zero. Compared with conventional loss functions such as mean squared error (MSE) or cross-entropy, the proposed loss better mitigates gradient explosion in long-distance predictions while maintaining a substantial penalty for large errors, thereby enhancing model stability and robustness. During training, the Adam optimizer is used in conjunction with a gradient clipping mechanism to prevent gradient explosion from disrupting parameter updates.

In summary, this module not only establishes a mapping from compressed feature representations to distance map outputs but also dynamically adjusts the predicted values using a task-specific loss function, thereby improving training stability and alignment with structural prediction objectives.

Finally, to ensure fair and consistent evaluation, all competing methods are assessed under identical input-feature configurations and data partitions. Specifically, models such as trRosetta, PDNet, MF-ProtDisMap, and SDP are executed using the uniformly generated input features of this study—including coevolutionary information, PSSMs, and conservation-based descriptors—while sharing the same training and testing datasets as MAKA-Map. This setup effectively eliminates the influence of feature discrepancies, ensuring that performance differences primarily reflect variations in model architecture and inference mechanisms. During evaluation, each model preserves its original network structure and parameter settings without modifications to its architecture or loss function. Evaluation metrics and computational procedures follow those in the respective original publications to maintain comparability and reproducibility. This protocol provides an objective assessment of the structural modeling capabilities of different models under uniform input conditions.

### 2.5. Performance Evaluation

To systematically evaluate the performance of the proposed MAKA-Map model in real-valued distance prediction, four widely used and effective evaluation metrics are employed in this study.

(1) Contact accuracy (precision)

To evaluate the model’s ability to identify residue pairs that are in spatial proximity based on the predicted real-valued distance maps, a threshold-based precision metric is employed, following common practice in contact map evaluation. Predicted distances are converted into binary contact predictions using a distance threshold of 8Å. The precision is defined in Equation (9).

(9) $$\mathrm{Precision}=\frac{\mathrm{TP}}{\mathrm{TP}+\mathrm{FP}},$$

where TP denotes the number of residue pairs correctly predicted to be in contact, while FP represents the number of pairs incorrectly predicted as contacts.

(2) Mean absolute error (MAE)

MAE is a primary metric used in regression tasks to quantify the average deviation between predicted and actual values. It is defined as the average of the absolute differences between the predicted and true distances, as shown in Equation (10):

(10) $$\mathrm{MAE}=\frac{1}{N}\sum_{i,j}\left|P_{ij}-Y_{ij}\right|,$$

where *i* and *j* denote the positions of two residues in the protein sequence, $P_{ij}$ is the predicted distance between residues *i* and *j*, and $Y_{ij}$ is the corresponding ground-truth distance, calculated as the Euclidean distance between their Cβ atoms in the PDB structure. The scalar *N* represents the total number of residue pairs that meet the evaluation criteria.

(3) Pearson correlation coefficient (*r*)

To further evaluate the model’s ability to capture the trend of the true distance distribution, the linear correlation between predicted and true values is quantified using the Pearson correlation coefficient, as defined in Equation (11):

(11) $$r=\frac{\sum_{i,j}\left(P_{ij}-\bar{P}\right)\left(Y_{ij}-\bar{Y}\right)}{\sqrt{\sum_{i,j}{\left(P_{ij}-\bar{P}\right)}^{2}}\sqrt{\sum_{i,j}{\left(Y_{ij}-\bar{Y}\right)}^{2}}},$$

where $\bar{P}$ and $\bar{Y}$ denote the mean predicted and mean true distances, respectively.

(4) Local Distance Difference Test (lDDT)

The lDDT metric [26] is used to evaluate the fidelity of local interatomic distances. It assesses the local geometric accuracy of the model by comparing predicted and reference inter-residue distances within a set of tolerance thresholds. Let T={0.5,1,2,4} denote the set of distance tolerances (in Å). The lDDT score is defined in Equation (12):

(12) $$\mathrm{lDDT}=\frac{1}{\left|T\right|}\sum_{t\in T}\frac{\sum_{i,j}I\left(\left|P_{ij}-Y_{ij}\right|<t\right)}{\sum_{i,j}1},$$

where $P_{ij}$ and $Y_{ij}$ represent the predicted and true distances between residues *i* and *j*, respectively, and I(·) denotes the indicator function, which returns 1 when the condition is satisfied and 0 otherwise.

(5) Matthews Correlation Coefficient (MCC)

To further evaluate the contact-level quality of the predicted distance maps, we derive binary contact maps from the real-valued distance predictions by applying an 8 Å distance threshold. The Matthews Correlation Coefficient (MCC) is then employed as a complementary evaluation metric, as it provides a balanced assessment that jointly considers true positives, true negatives, false positives, and false negatives, and is particularly suitable for highly imbalanced classification problems such as contact prediction [27]. The MCC is computed as follows:

(13) $$\mathrm{MCC}=\frac{TP\times TN-FP\times FN}{\sqrt{\left(TP+FP)(TP+FN)(TN+FP)(TN+FN\right)}},$$

where TP, TN, FP, and FN denote the numbers of true positives, true negatives, false positives, and false negatives, respectively.

### 2.6. Experimental Environment

The hardware and software environments for the experiments are summarized as follows:

The system is equipped with an AMD EPYC 7453 CPU (2.75 GHz base frequency), an NVIDIA GeForce RTX 3090 GPU (24.5 GB of VRAM, CUDA 12.0), and 60.1 GB of physical memory. The experiments were conducted on Ubuntu 20.04, using Python 3.10 as the programming language and PyTorch (https://pytorch.org/) as the deep learning framework for model development and evaluation.

## 3. Results

### 3.1. Comparison with Existing Real-Value Distance Structural Prediction Methods

To comprehensively evaluate the performance and generalization capability of MAKA-Map in real-valued distance prediction, benchmark experiments were conducted on three standard CASP datasets: CASP13, CASP14, and CASP15. The proposed model was compared with state-of-the-art methods, including trRosetta, PDNet, MF-ProtDisMap and SDP. Model evaluation employed two complementary metrics: (1) contact precision at multiple thresholds (L/5, L/2, and *L*) to assess the model’s ability to identify residue pairs in spatial proximity, and (2) mean absolute error (MAE) to quantify the accuracy of continuous distance predictions, thereby providing a comprehensive view of fine-grained predictive performance.

Under these challenging conditions, MAKA-Map consistently outperformed competing methods across multiple datasets. As shown in Table 1, on the CASP13 dataset, MAKA-Map achieved contact precisions of 86.53%, 76.01%, and 58.99% at thresholds of L/5, L/2, and *L*, respectively—substantially exceeding those of other methods. Correspondingly, MAKA-Map also yielded lower MAE values, achieving 1.3689 at the L/5 threshold—significantly outperforming PDNet (2.1573) and SDP (1.6960). For long-range predictions (*L*), the model achieved an MAE of 2.1019, significantly outperforming trRosetta (4.4342) and PDNet (3.2811), thereby validating its effectiveness in modeling long-range dependencies.

This favorable performance trend persisted on the more challenging CASP14 and CASP15 datasets. On CASP14, MAKA-Map achieved contact precisions of 85.44%, 75.25%, and 63.93% at the L/5, L/2, and *L* thresholds, respectively, while maintaining consistently low MAE values ranging from 1.5812 to 1.9755. On CASP15, although slightly trailing SDP in L/5 accuracy (82.77% vs. 84.52%), MAKA-Map outperformed SDP at the L/2 (74.24% vs. 74.03%) and *L* (59.35% vs. 58.39%) thresholds, while consistently achieving the lowest MAE values. These results further underscore the robustness of MAKA-Map in handling proteins with low homology and atypical structural features.

Prior studies have demonstrated that MCC, by jointly accounting for true positives, true negatives, false positives, and false negatives, provides a more reliable assessment of predicted contact maps than precision alone, particularly in low-homology and noisy prediction settings. As reported in Table 1, MAKA-Map achieves the highest MCC on both CASP13 and CASP14, with values of 0.5262 and 0.5197, respectively, indicating superior overall contact prediction quality on these challenging benchmarks. On CASP15, although SDP attains a slightly higher MCC (0.5305 vs. 0.5250), MAKA-Map remains highly competitive and consistently outperforms most competing methods. Notably, this marginal difference is accompanied by MAKA-Map achieving lower MAE values and stronger long-range (L/2 and *L*) contact precision, suggesting that its predicted contacts retain strong structural relevance. Taken together, these results demonstrate that MAKA-Map maintains robust and well-balanced contact prediction performance across diverse datasets, reinforcing its effectiveness under evaluation criteria that prioritize both accuracy and structural reliability.

Collectively, these results demonstrate that MAKA-Map’s performance advantage is not restricted to any particular dataset or difficulty level; instead, it consistently delivers stable and robust predictions across diverse challenges.

The observed robustness primarily stems from three key architectural innovations:

(1) Preserving residue-pair consistency throughout the encoding process to prevent structural information loss typically caused by conventional convolutional operations;
(2) Incorporating the Mamba module to enhance medium- and long-range residue interaction modeling, which is particularly beneficial for structurally complex targets;
(3) Integrating the Kolmogorov–Arnold Network (KAN) to improve local structural modeling through adaptive nonlinear transformations, thereby enhancing the representation of complex spatial topologies.

### 3.2. Analysis of the Effect of Long-Range Residues on Prediction

To further assess the models’ capacity to capture the global topology of real-valued distance maps, particular attention was paid to their performance on long-range residue pairs (LRPs). Unlike short- and medium-range contacts, LRPs impose stronger topological constraints, which are critical for maintaining structural integrity. However, their greater sequence separation and weaker coevolutionary signals present significant challenges for accurate prediction. Thus, the prediction of LRPs provides a more rigorous assessment of a model’s ability to capture complex long-range dependencies.

In this study, long-range residue pairs (LRPs) are defined using a sequence separation threshold of 24 residues or more (Seq-24), in accordance with established practices in the structural prediction literature. This threshold effectively minimizes local fragment bias and more accurately reflects the model’s capacity for global topological inference. Two evaluation metrics are employed for the filtered LRPs: (1) contact precision at varying sequence thresholds (L/5, L/2, and *L*), and (2) lDDT-Cβ, which quantifies local structural consistency.

As shown in Table 2, MAKA-Map exhibits superior performance across all evaluation metrics in the long-range contact prediction task on the CASP15 test set. At the L/5, L/2, and *L* sequence thresholds, MAKA achieves contact precisions of 0.7992, 0.6671, and 0.5023, respectively, outperforming trRosetta, PDNet, and SDP by varying margins. Notably, MAKA-Map attains an lDDT-Cβ score of 0.4544, which is substantially higher than that of PDNet (0.3620) and SDP (0.3360). These results indicate that MAKA-Map not only provides more accurate long-range contact predictions but also yields higher structural consistency and geometric plausibility in atomic-level reconstructions.

The performance gains of MAKA-Map can be primarily attributed to its architectural innovations, particularly the integration of the Mamba module and the Kolmogorov–Arnold Network (KAN). As a state space modeling component, Mamba provides robust global representation capabilities by efficiently propagating long-range information via its dynamic state-update mechanism. This design facilitates robust contextual integration, particularly in capturing dependencies among distantly located residues, thereby enhancing model stability and representational capacity. Complementing this, KAN introduces a spline-based nonlinear mapping framework that augments the model’s capacity to capture complex spatial interactions. By capturing fine-grained geometric features, KAN substantially improves the model’s discriminative capacity in long-range residue pair prediction. Together, these two components constitute a structurally complementary architecture that enhances MAKA-Map’s capacity to model remote topological patterns in real-valued distance maps.

Unlike traditional methods that heavily rely on strong local or evolutionary features and are often limited by sequence alignment quality or depth, MAKA-Map not only overcomes the bottleneck of local feature representation but also significantly improves the model’s ability to capture global structural consistency by integrating the architecturally complementary modeling mechanisms of Mamba and KAN. This capability has been consistently validated across multiple challenging benchmark datasets, further demonstrating MAKA-Map’s strong generalization performance and application potential in remote topological modeling.

### 3.3. Training Stability Under Five-Fold Cross-Validation

To verify the robustness of the adopted training protocol and to exclude potential bias introduced by a specific training–validation split, we evaluated training stability using five-fold cross-validation on the training dataset. Specifically, the complete training set was randomly partitioned into five mutually exclusive subsets. In each fold, four subsets were used for model training, while the remaining subset served as the validation set for model selection and early stopping. This process was repeated five times, ensuring that each subset was used as the validation set exactly once. Throughout the entire cross-validation procedure, the test set remained strictly independent and was not involved in any training, validation, or model selection steps. The test set consists of target proteins from CASP13, CASP14, and CASP15 and was used exclusively to evaluate the model’s generalization performance across different CASP tasks.

Under the five-fold cross-validation framework, performance metrics were computed on both the validation and test sets and reported as mean ± standard deviation (Mean ± Std) to quantify performance variability across folds. The Mean Absolute Error (MAE) on the validation set was 0.7327±0.0347, while the MAE on the comprehensive CASP test set was 0.8144±0.0384. The relatively small standard deviations indicate consistent model performance across different training subset partitions. Moreover, no individual fold exhibited systematically superior or inferior performance, suggesting the absence of dominant anomalous subsets or partition-induced biases that could unduly influence the overall evaluation.

In addition to regression accuracy, contact prediction performance was assessed on the CASP test set at different Top-*K* thresholds. The five-fold cross-validation results show that contact accuracy at the Top-L/5, Top-L/2, and Top-*L* levels reached 0.8496±0.0075, 0.7519±0.0121, and 0.6007±0.0179, respectively. These metrics exhibit limited variability across folds and display a monotonic decreasing trend as the Top-*K* threshold increases, which is consistent with commonly observed behavior in distance and contact prediction tasks. This further supports the stability and reliability of the model’s core predictive outcomes.

**Figure 5:**
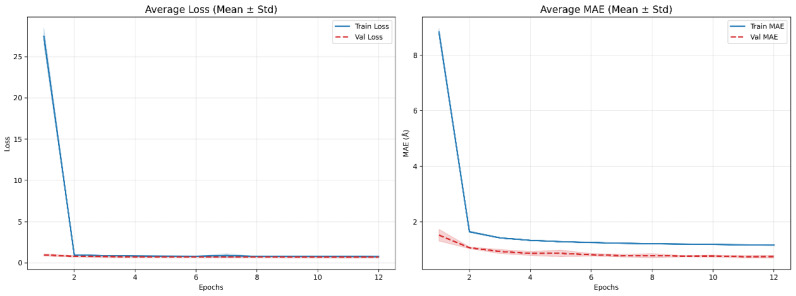
Training and validation loss and MAE curves under five-fold cross-validation.

Figure 5 presents the mean ± standard deviation curves of the training and validation losses, as well as the MAE values, across training iterations under the five-fold cross-validation setting. As shown in the figure, the model converges rapidly during the early training stages, after which both the loss and MAE on the training and validation sets gradually stabilize. Importantly, the validation curves do not exhibit sustained increases or notable divergence in later training stages, indicating that the model does not suffer from evident overfitting under the current training configuration.

It is worth noting that the MAE reported during cross-validation corresponds to a pixel-wise error computed over the predicted distance maps and is primarily used to monitor training convergence. Since this element-wise MAE is calculated across the entire distance map—including a large number of relatively easy-to-predict long-range residue pairs—its magnitude is typically lower than error metrics computed exclusively over critical prediction regions or Top-*K* residue pairs. Consequently, this MAE mainly reflects the model’s overall fitting behavior and training stability rather than fine-grained distance prediction accuracy on key residue pairs. This practice is consistent with commonly adopted training objectives in distance map-based deep learning methods. In contrast, the performance comparisons and analyses presented in this work focus on Top-*K*-based evaluation metrics on the independent test set. Although these two types of metrics differ in scope and emphasis, they provide complementary perspectives on training stability and final predictive performance.

In summary, the five-fold cross-validation results demonstrate that the proposed model exhibits robust training stability and consistent generalization across different training subset partitions. The training process converges smoothly, and prediction performance remains stable on the CASP13, CASP14, and CASP15 test sets, providing a reliable basis for evaluating the effectiveness of the proposed approach.

### 3.4. Ablation Experiments

To systematically evaluate the impact of input feature types and architectural components on real-valued inter-residue distance prediction in proteins, a series of ablation studies were conducted. Given the continuous nature of the prediction task, precision, mean absolute error (MAE), and the Pearson correlation coefficient (*r*) were employed as the primary evaluation metrics, capturing both the absolute deviation between predicted and ground-truth inter-residue distance maps and their overall linear correlation. These complementary metrics jointly reflect the model’s predictive accuracy and consistency from both numerical and statistical perspectives.

To assess the impact of feature inputs, two distinct feature combination schemes were constructed:

(1) CFP combination: comprising three classical feature types—co-evolutionary coupling scores (CCMpred), contact probability predictions (FreeContact), and position-specific scoring matrices (PSSM);
(2) AF combination: building upon the CFP features, contact potentials and Shannon entropy are additionally introduced, resulting in a total of five feature types.

The CFP feature set is widely used in protein structure modeling for capturing residue-level co-evolutionary and conservation signals. In contrast, contact potentials encode pairwise energy constraints that reflect structural stability, whereas Shannon entropy quantifies sequence variability and information content across multiple sequence alignments. These additional features are expected to enhance structural representation, especially in regions with limited co-evolutionary signals.

To analyze the contribution of each architectural component, four structural configurations were sequentially constructed:

(1) Baseline (Base): a residual backbone network without additional modules;
(2) KBL: the Kolmogorov–Arnold Network (KAN) module integrated on top of the Baseline;
(3) MBL: the Mamba module added to the Baseline architecture;
(4) MAKA: the full model integrating both KAN and Mamba modules.

**Figure 6:**
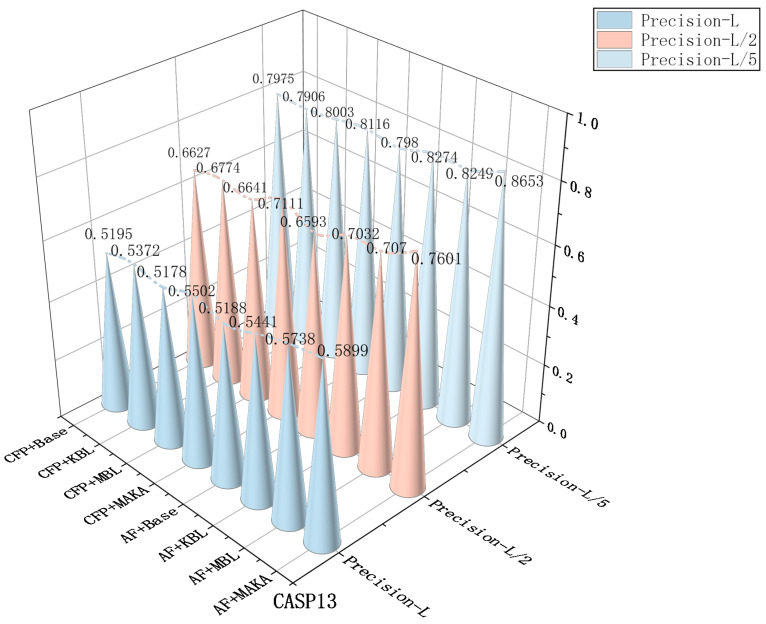
Comparison of Model Precision (L/5, L/2, L) for Baseline, KBL, MBL, and MAKA under CFP and AF Features at CASP13.

In Figure 6, under the CFP feature set, the Baseline model achieves precision scores of 0.7975 (L/5), 0.6627 (L/2), and 0.5195 (*L*), serving as a reference point for subsequent architectural enhancements. Incorporating the KAN module (KBL) improves performance at longer sequence separations, particularly at L/2 (0.6774) and *L* (0.5372), indicating an enhanced capacity for modeling long-range dependencies through nonlinear transformations. The integration of the Mamba module (MBL) leads to improved local accuracy (L/5: 0.8003), reflecting its effectiveness in capturing short-range structural regularities. The MAKA model, which combines both KAN and Mamba modules, achieves the best performance across all thresholds—0.8116 (L/5), 0.7111 (L/2), and 0.5502 (*L*)—confirming the complementary strengths of these modules in modeling both local and global dependencies.

With the AF feature set, which augments the CFP configuration by incorporating contact potentials and Shannon entropy, the overall performance is further enhanced. These enriched structural descriptors offer additional informative signals, particularly in regions characterized by sparse co-evolutionary data. The AF+MAKA architecture achieves the highest precision scores—0.8653 (L/5), 0.7601 (L/2), and 0.5899 (*L*)—highlighting the synergistic benefits of enriched input features and a dual-modality architectural design.

**Figure 7:**
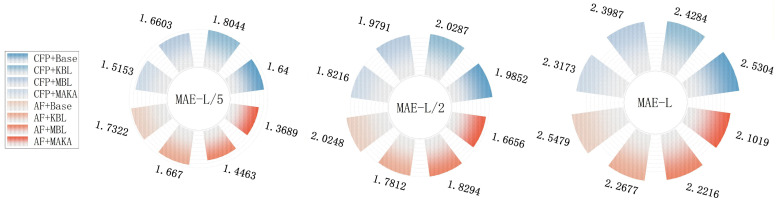
MAE Comparison (L/5, L/2, L) of Baseline, KBL, MBL, and MAKA Models between CFP and AF Features at CASP13.

**Figure 8:**
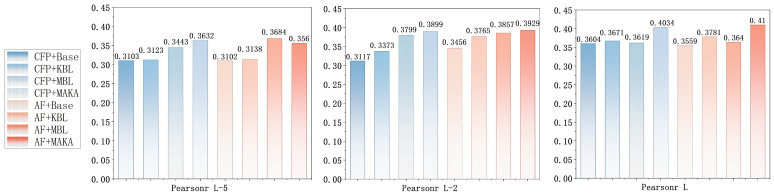
Pearson r Comparison (L/5, L/2, L) of Baseline, KBL, MBL, and MAKA Models between CFP and AF Features at CASP13.

Taking the *L*-level as an example for in-depth analysis, Figure 7 and Figure 8 further corroborate the above trend based on the MAE and Pearson correlation (*r*) metrics. Under the CFP configuration, the Baseline model achieves an MAE of 2.5304 and a Pearson *r* of 0.3604. Sequential integration of the KAN module (MAE: 2.4284; *r*: 0.3617) and the Mamba module (MAE: 2.3987; *r*: 0.3619) results in progressive performance improvements. The complete MAKA architecture achieves the lowest error (MAE: 2.3173) and the highest correlation (*r*: 0.4034), confirming the complementary contributions of both modules to regression accuracy (Figure 6).

Under the AF feature configuration, this trend is further accentuated. The MAKA model achieves the best overall performance, with the MAE reduced to 2.1019 and the Pearson correlation increased to 0.4100, as illustrated in Figure 7 and Figure 8. Similar performance trends were consistently observed at the L/5 and L/2 thresholds. The progressive integration of the KAN and Mamba modules leads to consistent improvements across all evaluation metrics and thresholds, culminating in optimal performance with the complete MAKA configuration.

Consistent trends are observed on CASP14 and CASP15 across Precision, MAE, and Pearson correlation (*r*) at the L/5, L/2, and *L* levels, as presented in Table 3, further validating the generalizability of the proposed approach. From the perspective of input features, although the co-evolutionary feature set—comprising CCMpred, FreeContact, and PSSM—provides robust structural signals, the addition of contact potentials and Shannon entropy introduces valuable complementary information. Specifically, these additional features enhance the model’s capacity to represent sequence diversity and to encode energy-based structural constraints. This is particularly beneficial in regions with sparse evolutionary information or long-range residue interactions, where the enriched feature set improves the model’s ability to infer contact strengths and spatial distribution patterns.

At the architectural level, the experimental results further confirm the functional complementarity between the two core modules. The KAN component enhances feature expressiveness through nonlinear transformations across channel dimensions, exhibiting clear advantages in modeling local spatial relationships. In contrast, the Mamba module, grounded in state space modeling, effectively captures global dependencies and cross-scale structural patterns. The synergistic integration of these two components enables MAKA-Map to maintain high predictive stability and accuracy across varying structural scales and residue densities.

Overall, the diversity of input features and the synergistic integration of structural modules constitute the core drivers of performance improvements in the MAKA-Map model. This study presents a scalable design framework within a lightweight architecture, offering a practical and generalizable model optimization paradigm for future real-valued distance prediction tasks.

### 3.5. Structural Relevance of MAKA-Map Predictions on CASP Targets

#### 3.5.1. Qualitative Analysis of Predicted Distance Maps on Representative CASP Targets

In the case study, three protein targets (T1041, T1078, and T1084) were selected for qualitative evaluation using the DISTEVAL tool [28]. These targets are representative of typical structural scenarios encountered in the test set.

For each target, the triangular region labeled **A** (left) corresponds to the ground-truth contact map derived from the experimental structure, whereas the triangular region labeled **B** (right) represents the contact map predicted by the MAKA-Map model.

**Figure 9:**
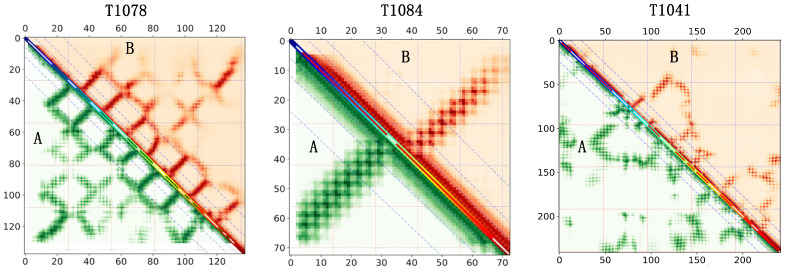
Visualisation of the predictive effect of the MAKA-Map model on three protein samples compared to the actual distance map.

(1) Sequence length and scale diversity.
  The three targets span noticeably different sequence lengths, including a short protein (T1084), a medium-length protein (T1078), and a longer protein (T1041). Variations in protein length are known to influence contact map sparsity, the relative abundance of long-range interactions, and the overall difficulty of distance prediction. Considering multiple length scales, therefore, helps to assess the general applicability of the proposed method.
(2) Diversity in contact map topology.
  The selected proteins exhibit clearly different contact map characteristics. In T1084, most contacts are concentrated near the main diagonal, which is typical for shorter or more regularly folded proteins dominated by local interactions. T1078, on the other hand, shows more evident block-like and repetitive off-diagonal patterns, suggesting more complex interactions between distant sequence segments or multiple structural units. As a longer protein, T1041 presents a comparatively sparser contact map with a higher proportion of long-range and cross-segment interactions, a pattern commonly observed in proteins with more complex structural organization. Together, these targets cover a range of contact distributions from predominantly local to strongly long-range interactions.
(3) Variation in prediction difficulty.
  As illustrated in Figure 9, the complexity of non-diagonal regions and the proportion of long-range contacts differ markedly among the three targets. For instance, T1041 contains more extensive interaction regions far from the diagonal, which generally corresponds to a more challenging prediction task. Consistent performance across such varying levels of difficulty provides evidence for the stability of the proposed approach.

As shown in Figure 9, the lower-left panels present the ground-truth inter-residue distance maps, whereas the upper triangular regions display the corresponding predictions generated by MAKA-Map. In some local regions, the predicted distances show slight shifts relative to the ground truth. These shifts are limited in extent and mainly reflect local misalignment rather than systematic errors.

At a broader scale, a closer inspection of the two left-most targets (T1041 and T1078) shows that the major strand–strand interactions are correctly captured. While some β-strand contacts appear slightly offset in register, the resulting shifts of the corresponding stripe patterns remain modest and do not affect the overall structural outlines or key topological features.

In contrast, for the third target, the predicted distance map shows close agreement with the ground truth across both local and long-range regions, with well-aligned stripe patterns and no noticeable register offset. This target therefore serves as a complementary case in which both local consistency and global organization are well preserved.

Overall, consistent fold-level organization is maintained across all three targets.

Taken together, these case studies demonstrate that MAKA-Map produces accurate real-valued inter-residue distance predictions at the fold level, with only minor local register variations.

#### 3.5.2. Three-Dimensional Structure Reconstruction Using MAKA-Map Predicted Distances

To further evaluate the structural relevance of the distance constraints predicted by MAKA-Map for downstream protein 3D structure prediction, we conducted structure reconstruction experiments on three independent test sets: CASP13, CASP14, and CASP15. Specifically, we used the distance maps predicted by MAKA-Map as constraint inputs to DistFold [29,30,31,32] and generated five independent 3D structural models for each protein target. For each model, we computed the TM-score and RMSD (root-mean-square deviation) [33] with respect to the corresponding native structure. TM-score primarily measures the correctness of the global topology, whereas RMSD quantifies the geometric deviation between the predicted and native structures. Both metrics are widely adopted in protein structure prediction and have clear physical interpretations.

Given the stochastic nature of conformational sampling in protein folding, our primary goal is to assess whether the predicted distance constraints are sufficiently informative to support at least one accurate structure reconstruction. Therefore, we adopted a best-of-five evaluation protocol. For each protein target, the Best TM-score is defined as the maximum TM-score among the five models, and the Best RMSD is defined as the minimum RMSD among the five models. In addition, to reflect the overall stability of the reconstruction process, we also report the Mean TM-score and Mean RMSD, computed by averaging the five models for each target. At the test-set level, all metrics are obtained by averaging the per-target statistics within each test set.

As summarized in Table 4, the Best TM-score on CASP13, CASP14, and CASP15 reaches 0.547, 0.586, and 0.573, respectively, consistently above 0.5. Since TM-score values above 0.5 typically indicate that the predicted structure captures the correct global fold, these results suggest that the MAKA-Map predicted distance constraints enable DistFold to reconstruct structures with reliable overall topology across all three test sets. Meanwhile, the Best RMSD values are 3.44 Å, 4.49 Å, and 5.25 Å for CASP13, CASP14, and CASP15, respectively. The gradual increase across successive CASP editions is consistent with the increasing difficulty and complexity of CASP targets, reflecting more challenging reconstruction conditions rather than instability of the proposed method.

**Figure 10:**
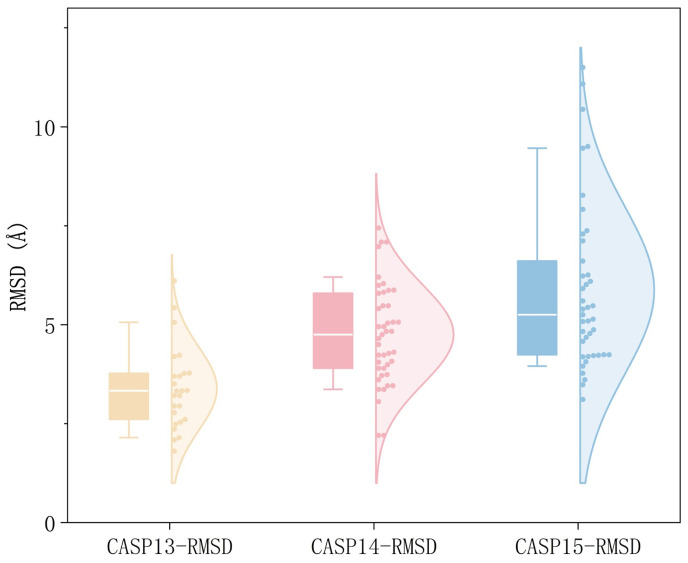
Distribution of per-target Best RMSD values on CASP13, CASP14, and CASP15.

To further characterize the reconstruction quality across test sets, we plotted the distributions of per-target Best RMSD values (Figure 10). The figure combines box plots and violin plots to visualize both the central tendency and the overall distribution of RMSD. The CASP13 distribution is relatively concentrated, indicating that the predicted constraints can reliably support accurate structure reconstruction on this test set. As the difficulty increases from CASP13 to CASP15, the Best RMSD distribution shifts toward higher values, while still exhibiting a reasonably concentrated pattern. Overall, these results indicate that although increased target complexity may reduce geometric accuracy to some extent, the distance constraints predicted by MAKA-Map remain structurally informative and usable across different CASP test sets.

Taken together, the above evidence demonstrates that three-dimensional reconstruction guided by MAKA-Map predicted distance information enables DistFold to consistently generate protein structure models with correct global topology on CASP13–15. The joint trends of Best TM-score and Best RMSD support that the predicted distance constraints are not only statistically consistent but also effective in practice for structure reconstruction. This observation is consistent with prior findings that a limited number of structurally informative contacts or distance constraints can be sufficient to reconstruct the overall protein fold, thereby validating the structural relevance of MAKA-Map predictions for downstream structural modeling tasks.

### 3.6. Comparison with AlphaFold3

AlphaFold3 (AF3) [34] excels at end-to-end 3D structure prediction, ensuring global topological consistency. However, its inferred distance maps often appear smoothed, as the model prioritizes geometric regularization over fine-grained pairwise details. In contrast, MAKA-Map explicitly learns inter-residue relationships, enabling it to capture sharper interaction signals that may be attenuated in coordinate-based reconstructions.

**Figure 11:**
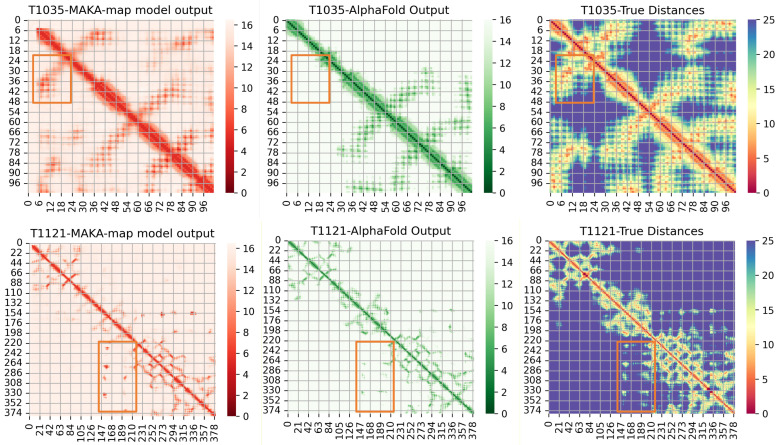
Visualisation of the predicted distance maps generated by MAKA-Map and AlphaFold in comparison with the ground-truth distance maps for two CASP targets (T1035 and T1121).

To elucidate this complementarity, we conducted a detailed visual analysis on targets T1035 and T1121 (Figure 11). For each target, the left panel shows the MAKA-Map prediction, the middle panel shows the AlphaFold-derived distance map, and the right panel shows the true $C_{\beta }$–$C_{\beta }$ distance map computed from the experimental structure. All panels are visualized using distance-based color scales (in Å), as indicated by the corresponding color bars. The boxed regions correspond to medium- to long-range structural features observed in the ground-truth distance maps and are used to facilitate visual comparison of the corresponding predicted patterns.

For T1035 (102 residues), the native distance map exhibits a dense off-diagonal interaction region. As highlighted by the orange boxes, MAKA-Map reproduces this region with higher contrast and more clearly defined boundaries, distinguishing contacting residue pairs more sharply than the more diffuse signals observed in the AF3-derived map. Nevertheless, closer inspection indicates that within these high-contrast regions, some fine-grained interaction patterns are slightly shifted relative to the native map, reflecting mild out-of-register effects in residue pairing. By comparison, although AF3 produces weaker signals in this region, the relative alignment of the corresponding interaction patterns is more consistent with the native configuration, benefiting from its stronger geometric constraints.

For T1121 (381 residues), the comparison reveals pronounced regional differences. MAKA-Map shows superior contrast in specific interaction modules, as indicated by the orange boxes, where interaction patterns are clearly resolved but appear attenuated in the AF3 prediction. Conversely, AF3 exhibits better performance in other densely interacting regions. In particular, within the region spanning residues 105–147 along the x-axis and 110–154 along the y-axis, AF3 more accurately reproduces the native contact patterns, whereas MAKA-Map shows reduced precision. In addition, a broader region spanning residues 105–210 (x-axis) and 132–198 (y-axis) lacks clear native-like signals in the predictions from both methods, indicating interaction regimes that remain challenging for both pixel-level and coordinate-based approaches.

Taken together, these case studies highlight a complementary trade-off between the two methods. MAKA-Map emphasizes interaction contrast and boundary definition for specific modules, while AF3 provides more reliable residue-level registration and better recovery in certain densely interacting regions. These observations suggest that explicitly learned distance representations can provide geometric information that is orthogonal to, and potentially synergistic with, end-to-end coordinate-based structure prediction.

### 3.7. Effect of MSA Depth on Prediction Reliability

To systematically evaluate the impact of multiple sequence alignment (MSA) depth and sequence diversity on prediction reliability, this study grouped test set targets based on the normalized metric Neff/L (effective number of sequences per residue), which jointly reflects MSA depth and sequence diversity.

We computed the effective number of sequences (NEFF) using NEFFy [35] with the similarity (sequence-identity) cutoff set to thr=0.8. In NEFFy, thr corresponds to the similarity cutoff (often denoted as θ) used for NEFF reweighting: two sequences are considered similar if their pairwise similarity/identity satisfies $S_{m,n}\geq \mathrm{thr}$ (i.e., $I[S_{m,n}\geq \mathrm{thr}]=1$), and redundancy among such similar sequences is down-weighted when aggregating sequence contributions. We chose thr=0.8 because it suppresses redundancy while maximally retaining subtle evolutionary signatures that are critical for real-valued distance map prediction. These fine-grained variations provide a more sensitive estimate of the MSA’s high-quality information depth and better match the MAKA-Map model’s ability to learn precise local geometric constraints at the Å scale.

For performance evaluation, two complementary metrics were selected. Precision L/5 was derived from real-valued distance predictions and computed by evaluating the top-ranked L/5 residue pairs under a contact threshold, thereby measuring the model’s ability to recover key structural constraints. Meanwhile, the lDDT metric was applied exclusively to long-range residue pairs (sequence separation ≥ 24) to assess local geometric consistency in the presence of non-local interactions. This metric combination simultaneously reflects the model’s performance in recovering short- to medium-range structural constraints and maintaining long-range topological consistency.

As summarized in Table 5, both Precision L/5 and long-range lDDT exhibit a clear upward trend with increasing Neff/L, indicating that deeper and more diverse MSAs generally provide more comprehensive evolutionary constraint information for structure prediction. When Neff/L exceeds 4, the model achieves substantially higher average performance on both Precision L/5 and lDDT, reflecting the positive impact of abundant evolutionary information on prediction accuracy.

Notably, even under conditions of shallow MSA depth (Neff/L < 1), where prediction performance declines compared to higher Neff/L ranges, the model maintains non-trivial levels of Precision L/5 and long-range lDDT. This observation indicates that the proposed method retains structural inference capability even with sparse evolutionary information or limited sequence diversity, partially mitigating the adverse effects of shallow MSAs on prediction reliability. This property is particularly relevant for orphan proteins or newly discovered proteins with scarce homologous sequences.

## 4. Conclusions

The MAKA-Map framework introduced in this study delivers a robust and highly effective approach for the prediction of real-valued inter-residue distances, a critical component for understanding protein structure and supporting downstream applications such as structural modeling, drug design, and protein engineering. By integrating the Mamba state space architecture with the Kolmogorov–Arnold Network (KAN) within a unified design, the MAKA-Map achieves a strong balance between capturing long-range dependencies and preserving the consistency between local features and global topology.

Specifically, the Mamba module advances global modeling by effectively capturing long-range inter-residue relationships across the protein, addressing one of the core challenges in structure prediction. In parallel, the KAN module strengthens the alignment between local representations and the overall structural context through its nonlinear, channel-wise transformation mechanism, allowing the model to encode fine-grained structural details while maintaining topological coherence. Together, these two components form a complementary design that significantly improves the quality of the resulting distance maps, yielding more accurate, topologically faithful, and structurally coherent predictions across a range of protein folds.

Extensive evaluations conducted on three standard CASP benchmark datasets (CASP13, CASP14, and CASP15) demonstrate that the MAKA-Map consistently outperforms existing state-of-the-art approaches, achieving notable improvements in long-range residue pair prediction and the modeling of complex topological constraints. In depth ablation studies and comparative analyses further highlight the pivotal role of both the Mamba and KAN modules, confirming their complementary contributions to enhancing prediction precision and structural representability.

Although the present work focuses primarily on the prediction of real-valued distance maps, the MAKA-Map framework provides a flexible foundation for future developments. Its architecture can be naturally extended to multi-task learning settings, supporting joint prediction of protein functional properties, molecular dynamics, or interaction interfaces. Such an approach could enable the seamless integration of structural and functional characterization within a single predictive model, opening new avenues for advances in protein engineering, molecular design, and other structure-driven biological applications.

## Figures and Tables

**Table 1:** Comparison of real-value distance prediction performance of different methods on CASP13, CASP14, and CASP15.

Dataset	Predictor	Precision			MAE			MCC
		L/5	L/2	L	L/5	L/2	L	
CASP13	trRosetta	0.7221	0.5802	0.4533	3.3817	3.7976	4.4342	0.3899
	pdnet	0.7537	0.6117	0.4860	2.1573	2.7109	3.2811	0.4080
	MF-ProtDisMap	0.8185	0.7285	0.5665	1.5932	1.9652	2.3629	0.4653
	sdp	0.7831	0.6861	0.5642	1.6960	1.9302	2.2350	0.4419
	MAKA	**0.8653**	**0.7601**	**0.5899**	**1.3689**	**1.6656**	**2.1019**	**0.5262**
CASP14	trRosetta	0.7271	0.6026	0.4945	2.9052	3.2271	3.7977	0.3734
	pdnet	0.7981	0.6962	0.5693	2.1241	2.5884	2.7834	0.3997
	MF-ProtDisMap	0.8077	0.7153	0.6190	1.6984	1.8987	2.4531	0.4814
	sdp	0.8342	0.7386	0.6131	1.5891	1.9932	2.1275	0.4972
	MAKA	**0.8544**	**0.7525**	**0.6393**	**1.5812**	**1.8603**	**1.9755**	**0.5197**
CASP15	trRosetta	0.7788	0.6718	0.5179	3.2162	3.3895	3.6178	0.3735
	pdnet	0.7895	0.6558	0.4958	2.2984	2.6933	2.9189	0.3790
	MF-ProtDisMap	0.8079	0.6979	0.5815	2.4698	2.5203	2.7987	0.5012
	sdp	**0.8452**	0.7403	0.5839	2.3012	2.4961	2.6157	**0.5305**
	MAKA	0.8277	**0.7424**	**0.5935**	**2.2186**	**2.4938**	**2.6145**	0.5250

**Table 2:** Performance comparison of different predictors under the Seq-24 condition on CASP13, CASP14, and CASP15 datasets.

Dataset	Predictor	Precision			lDDT
		L/5	L/2	L	
CASP13	trRosetta	0.6083	0.5006	0.4013	0.4000
	pdnet	0.6212	0.5543	0.4834	0.4058
	MF-ProtDisMap	0.7458	0.6358	0.5263	0.4513
	sdp	0.7211	0.6008	0.5069	0.3884
	MAKA	**0.7608**	**0.6775**	**0.5541**	**0.4836**
CASP14	trRosetta	0.6226	0.5018	0.4067	0.4329
	pdnet	0.7326	0.5933	0.4496	0.4188
	MF-ProtDisMap	0.7411	0.6077	0.4662	0.4243
	sdp	0.7665	0.6249	0.4822	0.3686
	MAKA	**0.7750**	**0.6594**	**0.5168**	**0.4976**
CASP15	trRosetta	0.7272	0.5906	0.4522	0.4510
	pdnet	0.7133	0.5663	0.4235	0.3620
	MF-ProtDisMap	0.7369	0.5815	0.4588	0.4445
	sdp	0.7988	0.6560	0.5005	0.3360
	MAKA	**0.7992**	**0.6671**	**0.5023**	**0.4544**

**Table 3:** Performance comparison of different model architectures and feature combinations on CASP14 and CASP15 datasets.

Dataset	Configuration	Precision			MAE			Pearson *r*		
		L/5	L/2	L	L/5	L/2	L	L/5	L/2	L
CASP14	CFP+Base	0.7889	0.6988	0.5677	2.0337	2.3759	2.7694	0.2049	0.2939	0.3424
	CFP+KBL	0.8030	0.7071	0.5729	1.8083	2.5569	2.8268	0.2145	0.3217	0.3577
	CFP+MBL	0.8146	0.7223	0.5816	1.9004	2.4208	2.6969	0.2208	0.3138	0.3423
	CFP+MAKA	0.8235	0.7196	0.6127	1.7245	2.0277	2.1543	0.2201	0.3309	0.3777
	AF+Base	0.8272	0.7232	0.5876	1.8649	2.1788	2.5398	0.2146	0.3076	0.3584
	AF+KBL	0.8322	0.7318	0.5937	1.6590	2.3449	2.2516	0.2245	0.3369	0.3747
	AF+MBL	0.8443	0.7492	0.6027	1.7435	2.2209	1.8742	0.2313	0.3283	0.3585
	AF+MAKA	**0.8544**	**0.7525**	**0.6393**	**1.5812**	**1.8603**	**1.9755**	**0.2305**	**0.3465**	**0.3954**
CASP15	CFP+Base	0.7500	0.6496	0.5150	4.0664	4.6219	4.9578	0.1487	0.2426	0.3707
	CFP+KBL	0.7632	0.6824	0.5371	3.5064	3.5935	3.8642	0.1597	0.2759	0.3602
	CFP+MBL	0.7925	0.6938	0.5564	3.7056	3.9139	3.5815	0.2276	0.3434	0.3777
	CFP+MAKA	0.7988	0.7168	0.5727	2.4183	2.7182	2.8498	0.2587	0.3574	0.3936
	AF+Base	0.7782	0.6732	0.5346	3.7306	4.2403	4.5475	0.1557	0.2538	0.3881
	AF+KBL	0.7910	0.7082	0.5575	3.2169	3.2968	3.5451	0.1671	0.2889	0.3769
	AF+MBL	0.8213	0.7190	0.5760	3.3996	3.5898	3.2858	0.2381	0.3596	0.3956
	AF+MAKA	**0.8277**	**0.7424**	**0.5935**	**2.2186**	**2.4938**	**2.6145**	**0.2708**	**0.3740**	**0.4121**

**Table 4:** Structure reconstruction performance on CASP test sets using DistFold guided by MAKA-Map predicted distance constraints.

Test Set	Best TM-Score	Best RMSD (Å)	Mean TM-Score	Mean RMSD (Å)
CASP13	0.547	3.44	0.528	4.94
CASP14	0.586	4.49	0.567	5.80
CASP15	0.573	5.25	0.551	7.07

**Table 5:** Effect of MSA depth (Neff/L) on prediction performance. Values are reported as mean ± standard deviation across targets within each Neff/L bin.

Neff/L Range	#Targets	Precision L/5	lDDT (Seq ≥ 24)
<1	8	0.512±0.327	0.343±0.117
1–2	11	0.755±0.228	0.410±0.114
2–4	7	0.735±0.181	0.394±0.163
>4	17	0.921±0.098	0.485±0.109

## Data Availability

We establish a webserver to implement the proposed method, which is currently accessible via https://bioinfor.nefu.edu.cn/MAKAMmap/ (accessed on 4 January 2026). Moreover, the source code and dataset for this study have been uploaded to https://github.com/Yuimynn/MAKA-map (accessed on 4 January 2026).

## References

[1] Wu H., Liu J., Jiang T., Zou Q., Qi S., Cui Z., Tiwari P., Ding Y. (2024). AttentionMGT-DTA: A multi-modal drug-target affinity prediction using graph transformer and attention mechanism. Neural Netw..

[2] Zhou C., Li Z., Song J., Xiang W. (2024). TransVAE-DTA: Transformer and variational autoencoder network for drug-target binding affinity prediction. Comput. Methods Programs Biomed..

[3] Jiang M., Li Z., Zhang S., Wang S., Wang X., Yuan Q., Wei Z. (2020). Drug-target affinity prediction using graph neural network and contact maps. RSC Adv..

[4] Shah P.M., Zhu H., Lu Z., Wang K., Tang J., Li M. (2025). DeepDTAGen: A multitask deep learning framework for drug-target affinity prediction and target-aware drugs generation. Nat. Commun..

[5] Wang J., Xiao Y., Shang X., Peng J. (2024). Predicting drug-target binding affinity with cross-scale graph contrastive learning. Briefings Bioinform..

[6] Kumar R., Romano J.D., Ritchie M.D. (2025). CASTER-DTA: Equivariant graph neural networks for predicting drug-target affinity. Briefings Bioinform..

[7] Berman H.M., Battistuz T., Bhat T.N., Bluhm W.F., Bourne P.E., Burkhardt K., Feng Z., Gilliland G.L., Iype L., Jain S. (2002). The Protein Data Bank. Acta Crystallogr. Sect. D.

[8] Zhang C., Zheng W., Mortuza S.M., Li Y., Zhang Y. (2020). DeepMSA: Constructing deep multiple sequence alignment to improve contact prediction and fold-recognition for distant-homology proteins. Bioinformatics.

[9] Li Y., Hu J., Zhang C., Yu D.J., Zhang Y. (2019). ResPRE: High-accuracy protein contact prediction by coupling precision matrix with deep residual neural networks. Bioinformatics.

[10] Madani M., Behzadi M.M., Song D., Ilies H.T., Tarakanova A. (2022). Improved inter-residue contact prediction via a hybrid generative model and dynamic loss function. Comput. Struct. Biotechnol. J..

[11] Zhao C., Wang S. (2024). AttCON: With better MSAs and attention mechanism for accurate protein contact map prediction. Comput. Biol. Med..

[12] Adhikari B. (2020). A fully open-source framework for deep learning protein real-valued distances. Sci. Rep..

[13] Yang J., Anishchenko I., Park H., Peng Z., Ovchinnikov S., Baker D. (2020). Improved protein structure prediction using predicted interresidue orientations. Proc. Natl. Acad. Sci. USA.

[14] Rahman J., Newton M.A.H., Ben Islam M.K., Sattar A. (2022). Enhancing protein inter-residue real distance prediction by scrutinising deep learning models. Sci. Rep..

[15] Zhang Y., Zhong S., Xu S., Wang Z., Xin C., Ni F., Yan F., Lu X., Sun S., Wang H. (2025). MF-ProtDisMap: Protein real-valued distance prediction with fusion of sequence and coevolutionary features. Int. J. Biol. Macromol..

[16] Liao W., Zhu Y., Wang X., Pan C., Wang Y., Ma L. (2024). LightM-UNet: Mamba Assists in Lightweight UNet for Medical Image Segmentation. arXiv.

[17] Li C., Liu X., Li W., Wang C., Liu H., Liu Y., Chen Z., Yuan Y. (2025). U-KAN Makes Strong Backbone for Medical Image Segmentation and Generation. Proc. AAAI Conf. Artif. Intell..

[18] Jones D.T., Kandathil S.M. (2018). High precision in protein contact prediction using fully convolutional neural networks and minimal sequence features. Bioinformatics.

[19] Kryshtafovych A., Schwede T., Topf M., Fidelis K., Moult J. (2019). Critical assessment of methods of protein structure prediction (CASP)—Round XIII. Proteins Struct. Funct. Bioinform..

[20] Kryshtafovych A., Schwede T., Topf M., Fidelis K., Moult J. (2021). Critical assessment of methods of protein structure prediction (CASP)—Round XIV. Proteins Struct. Funct. Bioinform..

[21] Kryshtafovych A., Schwede T., Topf M., Fidelis K., Moult J. (2023). Critical assessment of methods of protein structure prediction (CASP)—Round XV. Proteins Struct. Funct. Bioinform..

[22] Seemayer S., Gruber M., Soeding J. (2014). CCMpred-fast and precise prediction of protein residue-residue contacts from correlated mutations. Bioinformatics.

[23] Kajan L., Hopf T.A., Kalas M., Marks D.S., Rost B. (2014). FreeContact: Fast and free software for protein contact prediction from residue co-evolution. BMC Bioinform..

[24] Jones D.T., Singh T., Kosciolek T., Tetchner S. (2015). MetaPSICOV: Combining coevolution methods for accurate prediction of contacts and long range hydrogen bonding in proteins. Bioinformatics.

[25] Buchan D.W.A., Jones D.T. (2018). Improved protein contact predictions with the MetaPSICOV2 server in CASP12. Proteins-Struct. Funct. Bioinform..

[26] Mariani V., Biasini M., Barbato A., Schwede T. (2013). lDDT: A local superposition-free score for comparing protein structures and models using distance difference tests. Bioinformatics.

[27] Bhattacharya S., Bhattacharya D. (2020). Evaluating the significance of contact maps in low-homology protein modeling using contact-assisted threading. Sci. Rep..

[28] Adhikari B., Shrestha B., Bernardini M., Hou J., Lea J. (2021). DISTEVAL: A web server for evaluating predicted protein distances. BMC Bioinform..

[29] Bernardini M. (2021). DISTFOLD: Distance-guided Protein Folding. Master’s Thesis.

[30] Cheng J., Randall A., Sweredoski M., Baldi P. (2005). SCRATCH: A protein structure and structural feature prediction server. Nucleic Acids Res..

[31] Brünger A.T., Adams P.D., Clore G.M., DeLano W.L., Gros P., Grosse-Kunstleve R.W., Jiang J.S., Kuszewski J., Nilges M., Pannu N.S. (1998). Crystallography & NMR System: A New Software Suite for Macromolecular Structure Determination. Acta Crystallogr. Sect. D.

[32] Brunger A.T. (2007). Version 1.2 of the Crystallography and NMR system. Nat. Protoc..

[33] Sathyapriya R., Duarte J.M., Stehr H., Filippis I., Lappe M. (2009). Defining an Essence of Structure Determining Residue Contacts in Proteins. PLoS Comput. Biol..

[34] Abramson J., Adler J., Dunger J., Evans R., Green T., Pritzel A., Ronneberger O., Willmore L., Ballard A.J., Bambrick J. (2024). Accurate structure prediction of biomolecular interactions with AlphaFold 3. Nature.

[35] Haghani M., Bhattacharya D., Murali T.M. (2025). NEFFy: A Versatile Tool for Computing the Number of Effective Sequences. Bioinformatics.

